# Genistein Alleviates High-Fat Diet-Induced Obesity by Inhibiting the Process of Gluconeogenesis in Mice

**DOI:** 10.3390/nu14081551

**Published:** 2022-04-08

**Authors:** Mailin Gan, Xinquan Chen, Zongjian Chen, Lei Chen, Shunhua Zhang, Ye Zhao, Lili Niu, Xuewei Li, Linyuan Shen, Li Zhu

**Affiliations:** 1 Department of Animal Science, College of Animal Science and Technology, Sichuan Agricultural University, Chengdu 611130, China; ganmailin@stu.sicau.edu.cn (M.G.); 2020202018@stu.sicau.edu.cn (X.C.); chenzongjian@stu.sicau.edu.cn (Z.C.); chenlei815918@sicau.edu.cn (L.C.); 14081@sicau.edu.cn (S.Z.); zhye@sicau.edu.cn (Y.Z.); niulili@sicau.edu.cn (L.N.); xuewei.li@sicau.edu.cn (X.L.); shenlinyuan@sicau.edu.cn (L.S.); 2 Farm Animal Genetic Resource Exploration and Innovation Key Laboratory of Sichuan Province, Sichuan Agricultural University, Chengdu 611130, China

**Keywords:** genistein, gluconeogenesis, miR-451, *G6pc*

## Abstract

Genistein is an isoflavone phytoestrogen that has been shown to improve obesity; however, the underlying molecular mechanisms involved therein have not been clearly elucidated. In this study, we administered genistein to high-fat diet-induced obese mice to investigate its effect on hepatic gluconeogenesis. The results showed that genistein treatment significantly inhibited body weight gain, hyperglycemia, and adipose and hepatic lipid deposition in high-fat diet-induced obese mice. Glucose tolerance test (GTT), insulin tolerance test (ITT) and pyruvate tolerance test (PTT) showed that genistein treatment significantly inhibited gluconeogenesis and improved insulin resistance in obese mice. In addition, this study also found that genistein could promote the expression of miR-451 in vitro and in vivo, and the dual-luciferase reporter system showed that *G6pc* (glucose-6-phosphatase) may be a target gene of miR-451. Both genistein treatment and in vivo injection of miR-451 agomir significantly inhibited gluconeogenesis and inhibited the expression of *G6pc* and *Gk* (glycerol kinase, a known target gene of miR-451). In conclusion, genistein may inhibit gluconeogenesis in obese mice by regulating the expression of *Gk* and *G6pc* through miR-451. These results may provide insights into the functions of miR-451 and food-derived phytoestrogens in ameliorating and preventing gluconeogenesis-related diseases.

## 1. Introduction

With changes in dietary structure and lifestyle, the number of overweight and obese people continues to increase. According to estimates by the World Health Organization (WHO), by 2025, one in five adults worldwide will be obese [1]. Obesity is often accompanied by abnormal glucose metabolism and insulin resistance, and obesity is an important pathogenic factor of type 2 diabetes. In fact, most patients with type 2 diabetes are overweight or obese [2]. Diabetes is projected to be the seventh leading cause of death in the world by 2030 [3].

Insulin resistance and gluconeogenesis-induced abnormally elevated blood glucose are hallmarks of type 2 diabetes, but the underlying mechanisms leading to abnormal gluconeogenesis remain unclear [4,5]. Gluconeogenesis is the physiological process of synthesizing glucose from non-sugar substances, mainly in the liver. Under starvation conditions, gluconeogenesis can maintain blood glucose concentration and provide energy for highly glucose-dependent organs or tissues, such as the brain and red blood cells, which are important for the maintenance of normal physiological functions of the body [6]. However, persistent, high levels of gluconeogenesis are also a major cause of hyperglycemia in type 2 diabetes and severely impair insulin sensitivity [7]. The expression of gluconeogenic enzyme is regulated by a network of transcription factors and hormones. Rate-limiting enzymes involved in gluconeogenesis include pyruvate carboxylase (*Pcx*), phosphoenolpyruvate carboxykinase (*Pck1*), fructose-1,6-2 phosphatase (*Fbp*), and glucose-6-phosphate enzyme (*G6pc*) [8,9]. In addition, enzymes related to the production and transport of gluconeogenic substrates and some non-coding RNAs (ncRNA) were also found to be involved in the regulation of gluconeogenesis [10,11]. Regulation of these molecules can significantly improve hyperglycemia symptoms in animal models of diabetes [12]. Therefore, in-depth study of the regulation of hepatic gluconeogenesis may provide an effective target for the treatment of obesity and type 2 diabetes.

Most obesity drugs or methods have side effects such as easy rebound, affecting the nervous system and gastrointestinal dysfunction [13]. Therefore, finding a safe and effective way to lose weight has become a public health concern [14]. In recent years, dietary interventions or natural products have become a major research focus. Genistein is one of the most widely distributed food-borne phytoestrogens, its structure is similar to 17β-estradiol (E2), and it can show weak estrogenic or antiestrogenic activity in mammals [15]. The role of genistein in disease prevention has been widely reported, and it has beneficial effects in antitumor [16], anti-inflammatory [17], improve NAFLD [18], weight loss [19] and prevention of osteoporosis [20]. The function of genistein is usually related to its structure. However, in recent years, some researchers have found that genistein may act as a regulator of microRNAs (miRNAs) and participate in the regulation of anti-tumor [21], anti-depression [22], and alleviation of obesity [23] by regulating the expression of miRNAs. miRNAs are widely studied epigenetic regulators [24]. Previous studies have shown that miRNAs are involved in hepatic glucose and lipid metabolism [25,26], but there are still few reports on the regulation of gluconeogenesis by miRNAs. Meanwhile, it is unclear whether genistein regulates gluconeogenesis through miRNAs. In this study, we focused on the effect of genistein on hepatic gluconeogenesis in obese mice, and its potential mechanism of action.

## 2. Materials and Methods

### 2.1. Animals and Treatment

ICR mice (female, 8 weeks old) were purchased from Dashuo Laboratory Animal Co., Ltd. (Chengdu, China). All mice had free access to food and water during the experiment. Natural light cycle, ambient temperature: 22 °C ± 3 °C. During the experiment, mice were fed a normal maintenance diet (ND), a high-fat diet containing 40% fat (HFD), or HFD diet supplemented with 200 mg/kg genistein (HFD-G) [27]. The concentration of genistein in the short-term treatment group (HFD-G-7d) was 500 mg/kg in the diet. Genistein (Purity ≥ 98%) was purchased from Jingzhu Biotechnology (Nanjing, China). In vivo functional verification test of miR-451 was achieved by tail vein injection of miR-451 agomiR (cholesterol modification at the 3′ end, two thio skeleton modifications at the 5′ end, and four thio skeleton modifications at the 3′ end, full-chain methoxy modification, GenePharma, Shanghai, China). The injection dose of agomiR was 15 nmol/10 g (200 μg/10 g) body weight once every 2 days, and the index detection or sample collection was carried out after 1 week. Before the formal test, an obese mouse model (over 20% of the average body weight of the control group) was induced by high-fat diet. After the experiment, all mice were humanely euthanized by CO_2_ asphyxiation. Blood samples were collected and loaded into anticoagulant blood collection tubes (EDTA), centrifuged at 3000× *g* for 10 min, and the supernatant was taken.

### 2.2. Plasma Biochemical Index Test and Tissue Section

All biochemical parameters were determined using commercial kits (Nanjing Jiancheng Institute of Bioengineering, Nanjing, China). High-density lipoprotein cholesterol (HDL-C) and low-density lipoprotein cholesterol (LDL-C) levels were measured in plasma. Total cholesterol (TC), triglyceride (TG) and free fatty acid (FA) concentrations were measured in plasma and liver tissue. Referring to our previous study, changes in liver color were measured using a colorimeter (CR-300, Minolta, Japan) [27]. Liver tissue frozen sections were prepared and stained with hematoxylin-eosin (HE) and oil red.

### 2.3. Glucose Tolerance Test (GTT), Insulin Tolerance Test (ITT) and Pyruvate Tolerance Test (PTT)

Fasting strategies for mice differed in GTT, PTT, and ITT test trials [28]. After fasting for 16 h, mice were administered with glucose (2 g/kg body weight) or pyruvate (1.5 g/kg body weight) solution by intraperitoneal injection for GTT or PTT test. The ITT test was performed after the mice were fasted for 6 h, and insulin (0.75 U/kg body weight) was injected intraperitoneally. For all tests blood samples were drawn from the tail vein at 0, 15, 30, 60 and 120 min post dose. Blood glucose concentrations were measured using a glucometer (Accu-Chek Active, Roche, Ireland).

### 2.4. Cell Culture and Transfection

NCTC 1469 cells and HePG2 cells (Stem Cell Bank of Chinese Academy of Sciences, Beijing, China) were cultured in a carbon dioxide incubator (37 °C, 5% CO_2_). A 1 M solution of genistein was prepared using dimethyl sulfoxide (DMSO) under sterile conditions and stored at −20 °C [27]. NCTC1469 cells were seeded in 12-well plates. When the cell confluency reached about 50%, cells were stimulated with genistein (20 μM), and Lipofectamine 3000 (Invitrogen, Guangzhou, China) was used to transfect miR-451 mimic (miR-451 M), miR-451 inhibitor (miR-451 I) and negative control (NC, Ribobio, Guangzhou, China). After 24 h of treatment, the samples were lysed and collected according to the instructions (TRIzol reagent, Invitrogen, Guangzhou, China).

### 2.5. Real-Time Quantitative PCR

Total RNA was extracted from mouse liver (lysed after trituration with liquid nitrogen) and cells (directly lysed) using TRIzol reagent. Total RNA was reversed to cDNA using PrimeScript™ RT reagent Kit (Takara, Dalian, China), and cDNA was quantitatively detected by fluorescence on a CFX96 real-time PCR detection system (Bio-Rad, Richmond, CA, USA) using SYBR Premix Ex Taq II (2×) (Takara). Relative expression levels of mRNAs and miRNAs were calculated using the 2^−∆∆Ct^ method [29]. The primer sequences are listed in Supplementary Table S1.

### 2.6. Dual Luciferase Reporting System

The binding relationship between miRNA and target gene was verified using a dual-luciferase reporter system. A fragment of the 3′ UTR of *G6pc* containing the miR-451 target site was first amplified by PCR and inserted into the psiCHECK™-2 vector. For luciferase reporter assays, psiCHECK™-2 vector, miR-451M, NC or genistein were co-transfected into HePG2 cells using Lipofectamine 3000. After co-transfection, cells were harvested and lysed after 48 h of incubation. The fluorescence intensity of the lysate was measured using a dual-luciferase detection system (Promega, Madison, WI, USA). The relative fluorescence intensity was calculated according to the instructions.

### 2.7. Analysis of Liver Fatty Acids

After grinding the mouse liver tissue with liquid nitrogen, add 3 mL of n-hexane, invert and mix at 50 °C for 30 min, add 3 mL of methanol solution, and derivatize by shaking at 50 °C for 30 min. Add 1 mL of water and mix well. Leave the layers to stand still and take the upper layer for detection, (GC-MS 7890B-5977A, Agilent, Santa Clara, CA, USA).

### 2.8. Statistical Analysis

Results are presented as the mean ± sd. One-way analysis of variance was performed on more than two groups of samples using SPSS 22.0 software. Significance is marked in the form of superscript letters, with different superscript letters indicating significant difference (*p* ≤ 0.05), and superscript the same letter indicating insignificant difference (*p* > 0.05).

## 3. Results

### 3.1. Genistein Reduces Body Weight and Fat Deposition in Obese Mice

**Figure 1:**
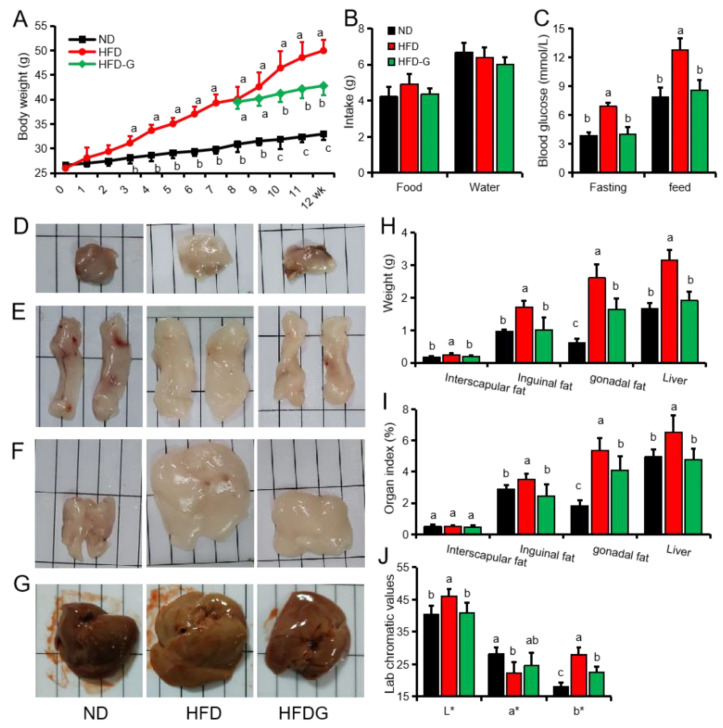
Genistein inhibits high-fat diet (HFD)-induced obesity in mice. (**A**) Weekly body weight of mice. ND: normal maintenance diet, HFDG: HFD diet supplemented with genistein. (**B**) Mean daily food and water intake. (**C**) Feed or fasting (8 h) blood glucose. (**D**–**G**) Images of the interscapular fat (**D**), inguinal fat (**E**), gonadal fat (**F**) and liver (**G**). (**H**) Weight of the interscapular fat, inguinal fat, gonadal fat and liver. (**I**) The organ index of the interscapular fat, inguinal fat, gonadal fat, and liver. (**J**) The chromatic aberration of the mice liver. *n* = 6. Different superscript letters indicate significant differences (*p* ≤ 0.05), and the same superscript letters indicate insignificant differences (*p* > 0.05). L* (0 is black, 100 is white); a* (positive and negative values are shown in red and green, respectively); b* (positive and negative values are shown in yellow and blue, respectively).

A mouse model of obesity was successfully established by feeding a high-fat diet (HFD) for 8 weeks (ND (normal maintenance diet): 30.88 ± 1.54 g; HFD: 39.79 ± 1.99 g). The weight of mice in the HFDG (HFD diet supplemented with genistein) group was significantly lower than that in the HFD group from the second week of feeding genistein, and the weight of the mice in the HFDG group increased by 3.28 g after one month of genistein treatment, while that in the HFD group increased by 9.90 g (Figure 1A). Genistein treatment did not affect the mice’s feed and water intake (Figure 1B). Fasting and feed blood glucose tests found that the HFD group was significantly higher than the ND and HFDG group (Figure 1C). Weighing of fat and liver tissue found that the weight of liver and in each part fat of the HFD group was significantly higher than that of the HFDG group and the ND group, and the organ indexes except the interscapular fat was also higher than those of the HFG group and the ND group (Figure 1D–I). However, only the gonadal fat was significantly higher in the HFDG group than in the ND group (Figure 1H,I). In addition, compared with the ND group, the liver tissue of the HFD group also showed a significant increase in L* (brightness), a significant decrease in a* (redness), and a significant increase in b* (yellowness). Compared with the HFD group, b* decreased significantly in the HFDG group (Figure 1J). These results indicate that genistein treatment significantly inhibited body weight gain and lipid deposition in adipose and liver tissue in obese mice.

### 3.2. Genistein Alleviates Insulin Resistance and Inhibits Gluconeogenesis in Obese Mice

**Figure 2:**
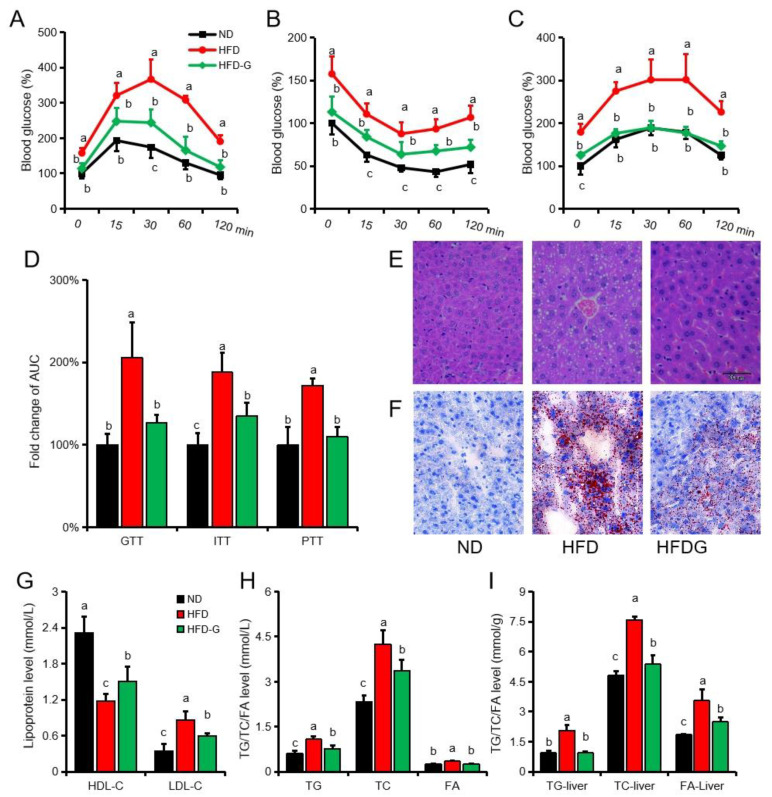
Genistein alleviates insulin resistance and hepatic lipid accumulation in obese mice. (**A**) GTT. (**B**) ITT. (**C**) PTT. (**D**) The area under the curve (AUC) of GTT, ITT and PTT. (**E**) HE staining of liver sections. (**F**) Oil Red O staining of liver sections (magnification, 200×). (**G**) Plasma HDL-C and LDL-C. (**H**) Plasma TG, TC and FA. (**I**) Triglycerides (TG), total cholesterol (TC) and free fatty acids (FA) in liver homogenate. *n* = 6. Different superscript letters indicate significant differences (*p* ≤ 0.05), and the same superscript letters indicate insignificant differences (*p* > 0.05).

To determine the effect of genistein on blood glucose metabolism in obese mice, GTT, ITT and PTT tests were performed (Figure 2A–C). Compared with the ND group, the blood glucose level in the HFD group was significantly increased at each time point, while the genistein treatment significantly alleviated the blood glucose increase caused by HFD (Figure 2D). HE staining of liver tissue showed that a large number of vacuoles appeared in hepatocytes after high-fat diet feeding, while the vacuoles were reduced but not completely disappeared after genistein treatment (Figure 2E). Oil red O staining showed that compared with the ND group, there were a large number of lipid droplets in the hepatocytes of the HFD group, while the size and number of lipid droplets in the HFDG group were between the ND group and the HFD group (Figure 2F). The detection of plasma and liver biochemical indexes showed that compared with the ND group, the HDL-C in the HFDG group was significantly lower than that in the ND group, while the plasma LDL-C, plasma TG, plasma TC, liver TC and liver FA was significantly higher than that in the ND group (Figure 2G–I). Meanwhile, compared with HFD group, HDL-C in HFDG group was significantly higher than that in the HFD group, while plasma LDL-C, plasma TG, plasma TC, liver TC and liver FA were significantly lower than that in the HFD group (Figure 2G–I). Plasma FA and liver TG of HDFG group was significantly lower than that of HFD group, but not significantly different from that of ND group (Figure 2H,I).

### 3.3. Genistein Regulates G6pc Expression via miR-451

**Figure 3:**
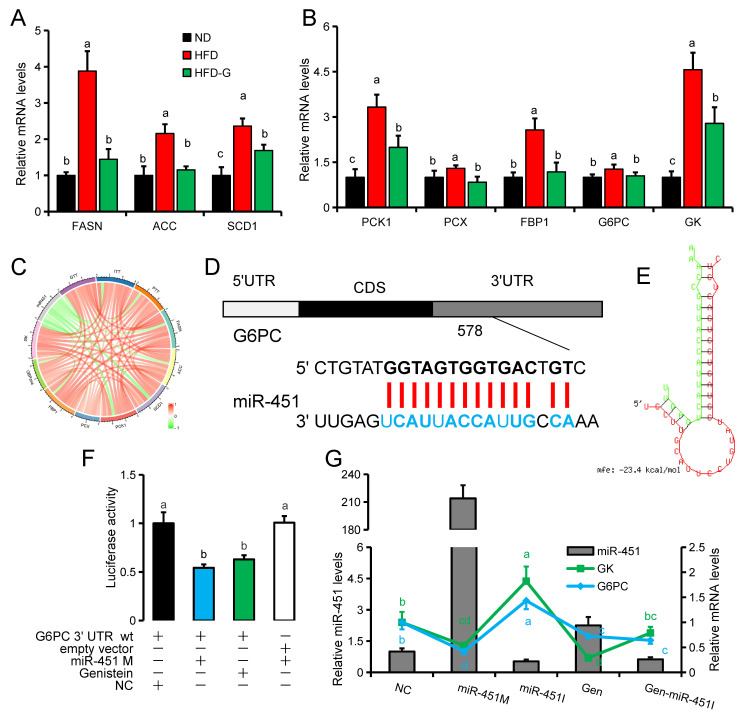
*G6pc* is a target gene of miR-451. (**A**) Expression of fat synthesis-related genes in liver, Fatty acid synthase (*Fasn*), Acetyl Co A carboxylase (*Acc1*), Stearoyl-CoAdesaturase-1 (*Scd1*). (**B**) Expression of gluconeogenesis-related genes in liver, pyruvate carboxylase (*Pcx*), phosphoenolpyruvate carboxykinase (*Pck1*), fructose-1,6-2 phosphatase (*Fbp*), and glucose-6-phosphate enzyme (*G6pc*), glycerol kinase (*Gk*). (**C**) Correlations between miR-451 and fat synthesis genes, gluconeogenesis genes, AUC-GTT, AUC-ITT and AUC-PTT. (**D**) Binding position of miR-451 to the 3′ UTR of *G6pc*. (**E**) Secondary structure of the binding site of miR-451 to the 3′ UTR of *G6pc*. (**F**) Fluorescence intensity of the dual-luciferase reporter assay system. (**G**) The expression of miR-451, *G6pc* and *Gk* in NCTC1469 cells after treatment with negative control (NC), miR-451 mimic (miR-451M), miR-451 inhibitor (miR-451I), 20 µM genistein (Gen) and 20 µM genistein + miR-451 inhibitor (Gen-miR-451I). (**A**,**B**) *n* = 6; (**F**,**G**) *n* = 3. Different superscript letters indicate significant differences (*p* ≤ 0.05), and the same superscript letters indicate insignificant differences (*p* > 0.05).

RT-qPCR results of fat synthesis (*Fasn, Acc1, Scd1*) and gluconeogenesis-related (*Pck1, Pcx, Fbp1, G6pc, Gk*) genes in mouse liver showed that high-fat diet feeding significantly increased the expression of these genes. The expression of all these genes was significantly decreased in the HFDG group compared with the HFD group (Figure 3A,B). Genistein has been reported to promote the expression of miR-451 in our previous study [27], and we speculated that the anti-obesity effect of genistein might be related to miR-451. Therefore, we further analyzed the correlation between miR-451 and the expression of these genes, and found that miR-451 was negatively correlated with the expression of fat synthesis and gluconeogenesis-related genes (Figure 3C). These results imply that miR-451 may play an important role in regulating hepatic fat synthesis and gluconeogenesis. Using RNAhybird [30] for sequence alignment and target gene prediction, we found that the 3’UTR of *G6pc* has a binding site for miR-451, and then we confirmed the binding relationship between the 3’UTR of *G6pc* and miR-451 using a dual-luciferase reporter system (Figure 3D–F). After transfection of miR-451 mimic (miR-451 M), miR-451 inhibitor (miR-451 M) or incubation with genistein (Gen) in NCTC1469 cells, genistein was found to have the ability to promote the expression of miR-451 in NCTC1469 cells (Figure 3G). Among different treatments, high expression of miR-451 corresponds to low expression of *G6pc* and *Gk* (Figure 3G).

### 3.4. Both Genistein and miR-451 Agomir Treatment Effectively Inhibit Hepatic Gluconeogenesis in Obese Mice In Vivo

**Figure 4:**
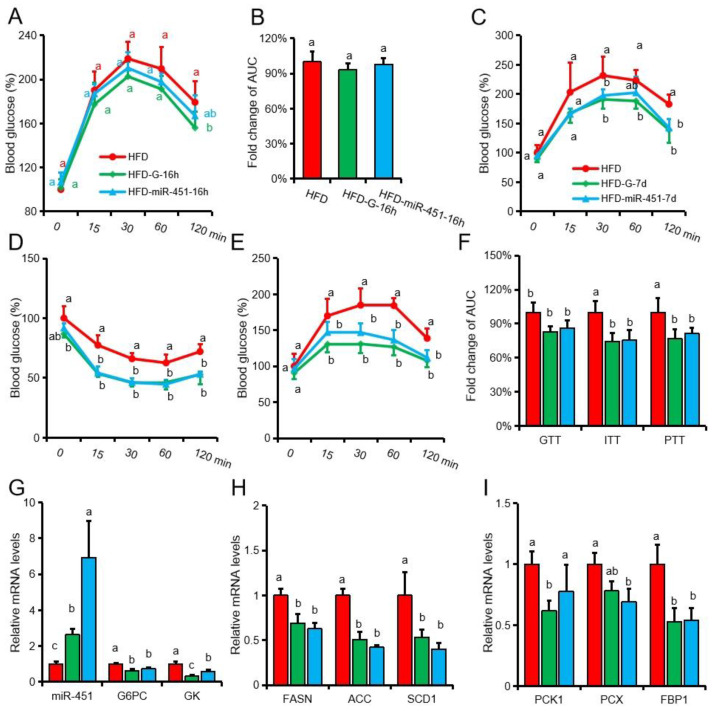
Both genistein and miR-451 agomir can effectively alleviate hepatic gluconeogenesis in obese mice. (**A**,**B**) Intraperitoneal PTT detection (**A**) and area under the curve (**B**) in obese mice, after genistein feeding for 1 day or injection of miR-451 agomir for 1 time. HFD-G-16h: Genistein supplemented in HFD diet and fed for 16 h, HFD-miR-451-16h: 16h after miR-451agomir injection in HFD diet group. (**C**–**F**). Intraperitoneal GTT (**C**), ITT (**D**), PTT (**E**) detection and area under the curve (**F**) in obese mice, after genistein feeding for 7 days or injection of miR-451 agomir 7 days. HFD-G-7d: Genistein supplemented in HFD diet and fed for 7 days, HFD-miR-451-7d: 7 days after miR-451agomir injection in HFD diet group. (**G**) Expression of miR-451 and its target genes *Gk* and *G6pc*. (**H**) Expression of fat synthesis-related genes in liver. (**B**) Expression of gluconeogenesis-related genes in liver. (**A**–**F**), *n* = 6; (**G**–**I**), *n* = 3. Different superscript letters indicate significant differences (*p* ≤ 0.05), and the same superscript letters indicate insignificant differences (*p* > 0.05).

We then examined the effects of genistein treatment for 1d and injection of miR-451 agomir once on PTT in obese mice, and found that the genistein-treated group was significantly lower than the HFD group, at only 120 minutes (Figure 4A). There were no significant differences in blood glucose in mice at other time points and in the total area under the curve (Figure 4B). The results showed that short-term treatment did not effectively improve gluconeogenesis in obese mice. Therefore, we extended the treatment time for 1 week and found that both miR-451 agomir and genistein were effective in reducing blood glucose levels in GTT, ITT and PTT tests in obese mice (Figure 4C–F). RT-qPCR showed that injection of miR-451 agomir significantly increased the level of miR-451 in mouse liver and significantly inhibited the expression of *G6pc*, *Gk*, *Fasn*, *Acc*, *Pcx* and *Fbp1* (Figure 4G–I). Genistein treatment solution significantly promoted the expression of miR-451 and inhibited the expression of *G6pc*, *Gk*, *Fasn*, *Acc*, *Pck1* and *Fbp1* (Figure 4G–I). These results suggest that genistein and miR-451agomir treatment for 1 week can effectively alleviate hepatic gluconeogenesis in obese mice.

### 3.5. Genistein and miR-451 Agomir Affect Mouse Liver Fatty Acid Composition

**Figure 5:**
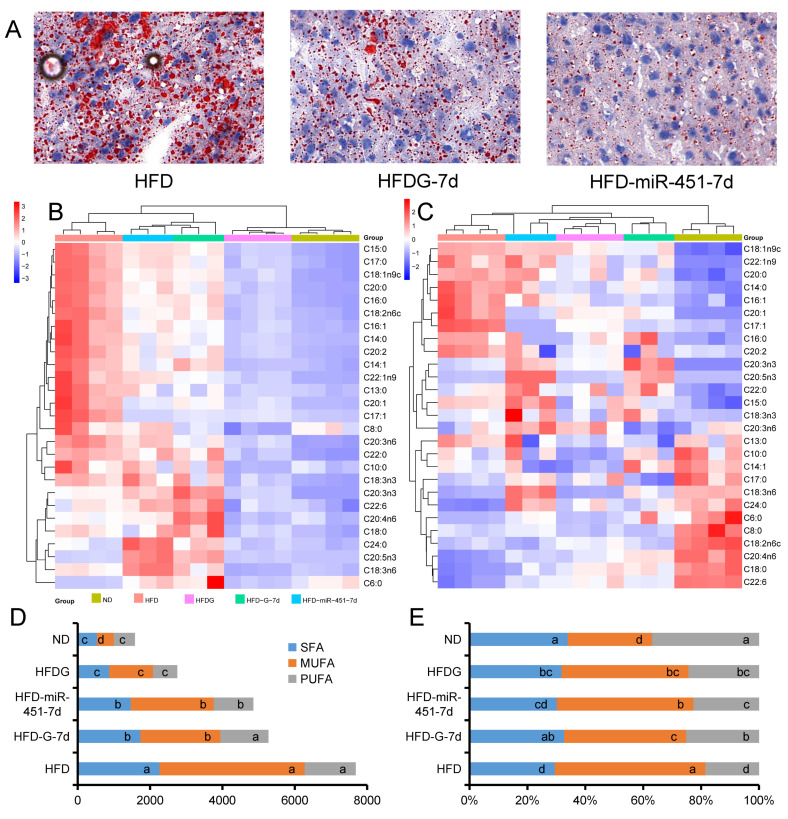
Effects of genistein and miR-451agomir on fatty acid content and composition in mouse liver. (**A**) Oil Red O staining of liver sections (magnification, 200×). (**B**) Cluster heatmap of the content of different fatty acids in the liver. (**C**) Cluster heatmap of different fatty acid composition of liver. (**D**) Saturated fatty acid (SFA), monounsaturated fatty acid (MUFA) and polyunsaturated fatty acid (PUFA) content in liver. (**E**) Composition of SFA, MUFA and PUFA in liver. The group of ND, HFD and HFDG, *n* = 4; the group of HFD-G-7d and HFD-miR-451-7d, *n* = 3. Different superscript letters indicate significant differences (*p* ≤ 0.05), and the same superscript letters indicate insignificant differences (*p* > 0.05).

Liver Oil Red O staining showed that genistein treatment and injection of miR-451 agomir 7d reduced the number and size of lipid droplets in the liver. Liver fatty acid content and composition was further analyzed (Figure 5A). In terms of content, the HFD group and the short-term treatment groups (HFD-G-7d and HFD-miR-451-7d) preferentially clustered into one clade, while the ND group and the long-term treatment group (HFD-G-7d) preferentially clustered into one clade (Figure 5B). In terms of fatty acid composition, different patterns were shown, showing that the 4 groups of mice fed a high-fat diet preferentially clustered into one branch, while the normal diet mice clustered into one branch alone (Figure 5C). The C18:1n9c, C22:1n9, C20:0, C14:0, C16:1, C20:1, C17:1, C16:0 and C20:2 ratios were increased in the liver of obese mice. The ratios of C18:1n9c, C22:1n9, C20:0, C14:0, C16:1, C20:1, C17:1, C16:0 and C20:2 were increased in the liver of obese mice, while C17:0, C18:3n6, C24:0, C6:0, C8:0, C18:2n6c, C20:4n6, C18:0 and C22:6 proportional reduction (Figure 5C). Saturated fatty acids (SFA), monounsaturated fatty acids (MUFA), and polyunsaturated fatty acids (PUFA) were further analyzed. Compared with the HFD group, the treatment groups (HFD-G, HFD-miR-451-7d and HFD-G-7d) had significantly lower SFA/MUFA and PUFA, except that the HFD-G-7d group had no significant effect on PUFA (Figure 5D). Fatty acid content was lower in both ND and HFDG groups (Figure 5B), but the content of monounsaturated fatty acids (MUFA) in the HFDG group was significantly higher than that in the ND group (Figure 5D). By analyzing the fatty acid composition, it was found that the HFD group was mainly characterized by increased MUFA and decreased PUFA, and different treatments could significantly alleviate this trend (Figure 5E).

## 4. Discussion

Genistein is a widely studied natural product, and its effects include anti-tumor, anti-inflammatory, antioxidant, and anti-osteoporosis [16]. In recent years, with the increase of obesity and type 2 diabetes, the research of genistein in resisting obesity and relieving diabetes has continued to emerge [31,32]. Hyperglycemia is one of the clinical features of diabetic patients, and while the continuous increase of gluconeogenesis is the main factor of hyperglycemia in diabetic patients, there are still few reports on the effect of genistein on gluconeogenesis [33]. Here, we demonstrate that genistein can partially lower blood sugar and improve insulin sensitivity by modulating gluconeogenesis. The hypoglycemic effect of genistein may be related to miR-451, *G6pc* and *Gk*.

In the present study, genistein significantly reduced lipid deposition in fat and liver in high-fat diet-induced obese mice. This is consistent with our previous observations [23]. In GTT, ITT and PTT experiments, genistein significantly improved glucose tolerance and insulin sensitivity, and significantly inhibited gluconeogenesis in obese mice. Many studies have found that genistein can improve blood glucose tolerance and insulin sensitivity, but there are fewer reports on its effects on gluconeogenesis [16]. miR-451 is a blood-cell-specific miRNA [34], not only involved in erythrocyte maturation [35], but also in glucose homeostasis [36], and our previous studies found that genistein can promote the expression of miR-451 in H9C2 [37], NCTC1469 and Raw246.7 cell lines [27], so we reasonably guessed that the hypoglycemic effect of genistein may be related to miR-451. Glycerol is one of the main raw materials for gluconeogenesis, and is produced by lipolysis of triglycerides in adipose tissue. After glycerol reaches the liver through blood circulation, it is phosphorylated by *Gk* (one of the target genes of miR-451 [36]) to produce glycerol 3-phosphate, which is further catalyzed by glycerol phosphate dehydrogenase to produce dihydroxyacetone phosphate and enter gluconeogenesis [38]. Notably, we also found and confirmed that *G6pc*, a key rate-limiting enzyme in gluconeogenesis [39], is also a target gene of miR-451. Zamani et al. also found that genistein could inhibit the expression of *Pck1* and *G6pase* [26]. Our results suggest that genistein can inhibit gluconeogenesis by regulating the expression of miR-451, but due to the special structure (molecular structure similar to E2), there may be other unknown regulatory mechanisms. In addition, because of its ability to bind to estrogen receptors, the possible side effects of genistein should also be fully considered, especially for hormone-sensitive organs and specific physiological states.

The liver is also the main site of fatty acid metabolism. On the one hand, a high-fat diet leads to the accumulation of exogenous fatty acids in the liver. At the same time, insulin resistance in obese mice leads to an increase in the synthesis of endogenous fatty acids in the liver [40]. In this study, *Fasn* and *Acc*, which are involved in fatty acid synthesis [41], were also found to be significantly increased in the liver of obese mice. In addition to participating in energy metabolism, fatty acids also play an important role in membrane structure and signal transduction, and different types of fatty acids play different roles [42]. The present study found that in addition to a significant increase in total fatty acid content in the liver of obese mice, fatty acid composition also changed (Table S2). High-fat diet-induced obese mice are characterized by increased MUFA and decreased PUFA. PUFA have important physiological functions [43]. Interestingly, by analyzing the content of SFA, MUFA and PUFA, we found that genistein treatment mainly reduced SFA and MUFA in liver after 1 week of treatment. In terms of composition, there was no significant difference in SFA, MUFA and PUFA between HFD-G group and HFD-G-7d group. These results suggest that genistein may exert beneficial physiological effects through PUFAs. In addition, the PUFA in the HFD-miR-451-7d group was significantly lower than that in the HFD-G group, suggesting that the effect of miR-451 on fatty acid composition in the liver of obese mice was different from that of genistein.

We also observed disrupted cholesterol homeostasis in obese mice. The main function of LDL-C in plasma is to supply blood cholesterol to cells, whereas the role of HDL-C is to transfer cholesterol from peripheral tissues to the liver [44]. Abnormal levels of plasma lipoproteins further exacerbate the disturbance of cholesterol metabolism. Corresponding to abnormal plasma lipoprotein levels was a marked increase in total cholesterol in the liver and plasma of obese mice. The level of HDL-C is negatively correlated with the occurrence of coronary heart disease. Elevated LDL-C and TG increase the risk of coronary heart disease [45]. Genistein can significantly inhibit LDL-C and up-regulate HDL-C, suggesting it has application potential in the treatment of cardiovascular diseases.

## 5. Conclusions

In conclusion, our study demonstrated the improvement effect of genistein on hepatic glucose and lipid metabolism in obese mice. Genistein can alter liver fatty acid content and composition to reduce hepatic lipid deposition and alleviate hyperglycemia symptoms by inhibiting hepatic gluconeogenesis. miR-451 and its target genes *Gk* and *G6pc* may play an important role in the inhibition of hepatic gluconeogenesis by genistein in obese mice. These results provide a reference for understanding the potential regulatory mechanism of genistein in improving obesity.

## Data Availability

If additional data related to this study are required, please consult the corresponding authors.

## Supplementary Materials

The following supporting information can be downloaded at: https://www.mdpi.com/article/10.3390/nu14081551/s1, Table S1: Sequences of PCR primers used in this study; Table S2: Liver fatty acid composition in mice.
